# Bone Morphogenetic Protein (BMP)-4 and BMP-7 regulate differentially Transforming Growth Factor (TGF)-β1 in normal human lung fibroblasts (NHLF)

**DOI:** 10.1186/1465-9921-11-85

**Published:** 2010-06-23

**Authors:** Sophie Pegorier, Gaynor A Campbell, A Barry Kay, Clare M Lloyd

**Affiliations:** 1 Leukocyte Biology Section, MRC and Asthma UK Centre in Allergic Mechanisms of Asthma, National Heart and Lung Institute, Faculty of Medicine, Imperial College London, London, UK

## Abstract

**Background:**

Airway remodelling is thought to be under the control of a complex group of molecules belonging to the Transforming Growth Factor **(**TGF)-superfamily. The Bone Morphogenetic Proteins **(**BMPs) belong to this family and have been shown to regulate fibrosis in kidney and liver diseases. However, the role of BMPs in lung remodelling remains unclear. BMPs may regulate tissue remodelling in asthma by controlling TGF-β-induced profibrotic functions in lung fibroblasts.

**Methods:**

Cell cultures were exposed to TGF-β1 alone or in the presence of BMP-4 or BMP-7; control cultures were exposed to medium only. Cell proliferation was assessed by quantification of the incorporation of [3H]-thymidine. The expression of the mRNA encoding collagen type I and IV, tenascin C and fibronectin in normal human lung fibroblasts (NHLF) was determined by real-time quantitative PCR and the main results were confirmed by ELISA. Cell differentiation was determined by the analysis of the expression of α-smooth muscle actin (α-SMA) by western blot and immunohistochemistry. The effect on matrix metalloproteinase (MMP) activity was assessed by zymography.

**Results:**

We have demonstrated TGF-β1 induced upregulation of mRNAs encoding the extracellular matrix proteins, tenascin C, fibronectin and collagen type I and IV when compared to unstimulated NHLF, and confirmed these results at the protein level. BMP-4, but not BMP-7, reduced TGF-β1-induced extracellular matrix protein production. TGF-β1 induced an increase in the activity of the pro-form of MMP-2 which was inhibited by BMP-7 but not BMP-4. Both BMP-4 and BMP-7 downregulated TGF-β1-induced MMP-13 release compared to untreated and TGF-β1-treated cells. TGF-β1 also induced a myofibroblast-like transformation which was partially inhibited by BMP-7 but not BMP-4.

**Conclusions:**

Our study suggests that some regulatory properties of BMP-7 may be tissue or cell type specific and unveil a potential regulatory role for BMP-4 in the regulation of lung fibroblast function.

## Background

Asthma is a chronic inflammatory disorder of the airways characterized by structural changes of the airway wall, collectively named remodelling. Airway remodelling is characterized by subepithelial fibrosis, with thickening of the subepithelial basement membrane, fibroblast and myofibroblast accumulation, increased expression of fibrogenic growth factors, and augmented extracellular matrix (ECM) deposition in the subepithelial areas of the proximal airways [1-3]. Other features of airway remodelling include an increase in airway smooth muscle (ASM) mass caused by hypertrophy and hyperplasia, goblet cell hyperplasia, and angiogenesis [1-3]. Resident lung fibroblasts and myofibroblasts are the primary source of ECM proteins which are released under the influence of growth factors such as Transforming Growth Factor **(**TGF)-β superfamily members [4,5].

The TGF-β superfamily of ligands comprises more than 35 members in mammals, including TGF-β_1-3_, activins and Bone Morphogenetic Proteins **(**BMPs), which are the largest subgroup of structurally and functionally related proteins of this family [6]. TGF-β contributes to airway remodelling in asthma via induction of a multitude of responses in lung resident cells. These include apoptosis of epithelial cells, dysregulation of epithelial cell adhesion properties leading to damage of the epithelial cell layer [7], and enhancement of goblet cell proliferation and mucus hyper-secretion [5,8]. TGF-β also induces differentiation of fibroblasts into myofibroblasts and their subsequent proliferation, as well as collagen and other ECM protein production including tenascin-C (Tn-C) and fibronectin by these cells [9-11]. Tn-C is a purported marker of reactivation of the epithelial-mesenchymal trophic unit (EMTU) in asthma. Transient increase of Tn-C in the asthmatic airway following allergen challenge has been identified [12], and increased production of fibronectin by myofibroblasts may promote epithelial-mesenchymal transition *in-vivo *[13]. TGF-β also enhances proliferation of ASM cells and contributes to increased ASM mass [14,15]. Anti-TGF-β treatment has been found to prevent these airway remodelling changes in a murine model of chronic allergen challenge model [8,16].

The BMPs are a large class of multifunctional growth factors and are a major developmental signalling pathway critical for embryogenesis and tissue generation in organs such as the kidney and lung [17]. However, they are also essential during postnatal life, and regulate cell proliferation, differentiation, apoptosis, angiogenesis, and secretion of ECM components [17,18]. BMP-7 is thought to have inhibitory effects since it is able to counteract TGF-β1-induced fibrotic effects *in vitro *and to reverse established fibrosis in organs as diverse as the kidney, heart and colon [19-26]. However, these antifibrotic effects may be tissue and indeed cell specific since BMP-7 has no effect in a bleomycin-induced lung fibrosis model or on skin fibrosis [27], and does not reverse TGF-β1-induced epithelial-to-mesenchymal transition in human renal proximal tubule epithelial cells [28]. In contrast, little is known about the role of BMP-4 *in vitro *or *in vivo *in lung remodelling although previous studies have shown that BMP-4 inhibits proliferation and promotes myocyte differentiation of lung fibroblasts [29,30]. We recently demonstrated for the first time the presence of BMP-4 and BMP-7 as well as their receptors in the airways of adult asthmatics [31]. In this study, BMP receptor expression was down-regulated in asthmatic airways compared to healthy controls which may impede repair responses, although allergen provocation increased expression of BMP-7, activated BMP signalling and increased receptor expression in the asthmatic airways, all of which may contribute to repair [31]. The cellular targets and regulatory mechanisms activated by the BMPs remain to be determined and nothing is known about their function in the adult lung.

We hypothesised that BMP-4 and BMP-7 may regulate airway remodelling by inhibiting TGF-β1 effects in lung fibroblasts. Our results indicate that BMP-4, but not BMP-7, inhibits TGF-β1 induced cell proliferation of normal human lung fibroblasts (NHLF) and also blocks the production of ECM proteins by these cells. Both BMP-4 and BMP-7 inhibited the differentiation of fibroblasts into myofibroblasts and blocked the release of matrix metalloproteinase (MMP)-13, whereas only BMP-7 was able to inhibit TGF-β1-induced MMP-2 activity. In conclusion, BMP-4 acts as a potent negative regulator of TGF-β1 whereas BMP-7 is only partially effective in our *in vitro *model of fibroblast activation.

## Methods

### Normal human lung fibroblast culture and stimulation

Primary adult human lung fibroblasts obtained from healthy, non-smoking donors, (NHLF, Lonza Rockland Inc, Rockland, ME, USA) were seeded in 12-well plastic culture dishes (Sigma-Aldrich, Gillingham, Dorset, UK) and grown at 37°C in a humidified 5% CO_2 _atmosphere in fibroblast growth medium (FGM, Lonza Rockland Inc, Rockland, ME, USA) supplemented with 0.5 ml recombinant human fibroblast growth factor-B, 0.5 ml insulin, 0.5 ml gentamicin sulphate amphotericin-B and 2% foetal bovine serum (FBS). Once they reached 80% confluence, NHLF were stimulated for 24 h, 48 h and 72 h with either 5 ng/ml TGF-β1 or 100 ng/ml human recombinant BMP-4 or BMP-7 (R&D Systems Europe Ltd., Abingdon, UK). Cells were also stimulated with 5 ng/ml TGF-β1 in combination with either 100 ng/ml BMP-4 or BMP-7. Those concentrations are based on previously published data obtained in other cell types [24,32]

### Assessment of NHLF viability and proliferation

The effect of TGF-β1 and BMPs on NHLF viability was determined by colorimetric MTT based assay (Cell Proliferation Kit I [MTT]; Roche Diagnostics Ltd, West Sussex, UK) according to the manufacturer's instructions. Briefly, NHLF were seeded in 96-well plates (Sigma-Aldrich, Dorset, UK) and stimulated as described above for 24, 48, and 72 h in FGM with or without 2% FBS. Cells were labelled by 4 h incubation in MTT labelling agent at 37°C and then solubilisation solution was added overnight. The plates were read on a Microplate reader photometer at 600-nm wavelength. Three independent experiments were conducted. For proliferation experiments, fibroblasts were stimulated as above for 36 h with addition of [3H]-thymidine (1 μCi/ml) for the final 6 h of incubation. Incorporation of [3H]-thymidine was terminated by washing the cells twice with PBS. Cells were then lysed with 0.1 N NaOH, and radioactivity (degradation/minute) measured by a scintillation counter and used as an index of DNA synthesis and fibroblast proliferation, five independent experiments were conducted.

### RNA isolation and reverse transcription

Confluent NHLF that had been stimulated for 24 h were recovered in 350 μl lysis buffer RLT contained in the RNeasy Mini Kit (Qiagen, West Sussex, UK) supplemented with 1% 2-βmercaptoethanol (Sigma-Aldrich, Gillingham, Dorset, UK) and then stored at -80°C. Total RNA was isolated using this same kit according to manufacturer's instructions. Reverse transcription was performed for 2 h at 37°C using Moloney murine leukemia virus reverse transcriptase (Promega UK, Southampton, UK) and 1 μg total RNA in 50 μl volume.

### Real-time quantitative PCR

Real-time quantitative PCR was performed using the SYBRGreen JumpStart *Taq *Ready Mix detection kit (Sigma-Aldrich, Gillingham, Dorset, UK). In all assays, cDNA was amplified using a standardized program (2 min JumpStart *Taq *Polymerase activation step at 94°C; 40 cycles of 30 s at 94°C and 1 min at 60°C). All assays were performed in a volume of 20 μl, and primers were used at a final concentration of 0.33 μM. Reactions were conducted using the PCR ABI 7500 apparatus (Applied Biosystems, Warrington, UK). For a more accurate and reliable normalization of the results, the intensity of gene expression was normalized to the geometrical mean of the levels of transcripts encoding the 3 most stable housekeeping genes: ubiquitin-C (*UBC*), succinate dehydrogenase (*SDHA*), and ribosomal protein 13a (*RPL13a*) [33]. Normalization and calculation were assessed using the GeNorm method [33]. Primers were designed using Primer Express 2 Software (Applied Biosystems, Warrington, UK) and were synthesized by Invitrogen Life Technologies Ltd. (Paisley, UK). Primer sequences and basal gene expression in unstimulated NHLF are described in Table 1.

**Table 1:** Real-time primer sequences and basal levels of transcript expression in normal human lung fibroblasts

GenBank Identifier	Gene	Forward Primer	Reverse Primer	Basal Ct
NM_001105	ALK-2	CGGGAGATGACCTGTAAGACCCCG	GGGCCGTGATGTTCCTGTTAC	25.00 ± 0.70
NM_004329	ALK-3	CAGAAACCTATTTGTTCATCATTTCTCG	ATCCCAGTGCCATGAAGCATAC	21.97 ± 0.82
NM_001203	ALK-6	CGAATGGGGTGTAGGTCTTTATTACATTCG	CCCATTCCTCATCAAAGAAGATCA	26.50 ± 0.93
NM_001204	BMPRII	CGGTTTCCACCTCATTCATTTAACCG	ACAGAGACTGATGCCAAAGCAAT	24.93 ± 0.42
NM_000088	COL1a1	CTTTGCATTCATCTCTCAAACTTAGTTTT	CCCCGCATGGGTCTTCA	19.03 ± 0.69
NM_001845	COL4a1	CTAATCACAAACTGAATGACTTGACTTCA	AAATGGCCCGAATGTGCTTA	19.87 ± 0.95
X02761	Fibronectin	TGGACCAGAGATCTTGGATGTTC	CGCCTAAAACCATGTTCCTCAA	21.70 ± 0.79
X56160	Tenascin C	GGTCCACACCTGGGCATTT	TTGCTGAATCAAACAACAAAACAGA	17.00 ± 0.92
NM_001613	αSMA	CCGACCGAATGCAGAAGGA	ACAGAGTATTTGCGCTCCGAA	20.60 ± 0.10
NM_021009	UBC	CACTTGGTCCTGCGCTTGA	TTTTTTGGGAATGCAACAACTTT	17.50 ± 1.35
NM_012423	RPL13A	CCTGGAGGAGAAGAGGAAAGAGA	TTGAGGACCTCTGTGTATTTGTCAA	19.65 ± 0.31
NM_004168	SDHA	TGTGTCCATGTCATAACTGTCTTCATA	AAGAATGAAGCAAGGGACAAAGG	19.00 ± 0.91

### Determination of total soluble collagen, tenascin C and fibronectin in cell supernatant

The levels of total soluble collagen, tenascin C and fibronectin were assessed in supernatants from NHLF stimulated for 48 h, and 72 h with TGF-β1 and BMP-4 or BMP-7 as described. Soluble collagen was measured by Sircol assay (Biocolor Ltd., County Antrim, UK) and tenascin C and fibronectin by ELISA (Human Tenascin-C Large kit from Immuno-Biological Laboratories, Gunma, Japan and Fibronectin ELISA reagent kit from Technoclone Ltd., Surrey, UK). The threshold of detection was 2.5 μg/ml for total soluble collagen, 0.38 ng/ml for tenascin C and 250 ng/ml for fibronectin.

### MMP activation and production

MMP-1 and MMP-2 activation was quantified by gelatin zymography. Proteins of cell supernatants were separated on a 10% acrylamide/0.1% gelatin gel (Invitrogen Life Technologies Ltd., Paisley, UK). After electrophoresis, the gel was washed twice for 30 min in a buffer containing 2.7% Triton X-100 at room temperature and incubated for 48 h in 50 mM Tris-base, 40 mM HCl, 200 mM NaCl, 5 mM CaCl_2_, 0.02% Brij 35, at 37°C. The gels were then stained with Coomassie brilliant blue and analysed. Bands were quantified by densitometry with ImageJ software. Levels of MMP-13 were quantified in supernatants from NHLF stimulated for 72 h by ELISA (Collagenase-3 ELISA Kit from Merck Chemicals Ltd. Nottingham, UK). The threshold of detection was 32 pg/ml.

### αSMA immunostaining

To determine whether BMPs can counteract TGF-β1-induced myofibroblast formation, NHLF were grown on chamber slides (ICN, Basingstoke, U.K) for 3 days until ~70% confluent and cells were stimulated as described above for 72 h, washed with PBS and fixed with 4% paraformaldehyde. Following permeabilization in PBS containing 0.1% saponin, endogenous peroxidases were removed by 45 min incubation in peroxidase blocking solution (DAKO, Glostrup, Denmark) and avidin and biotin were blocked using the avidin/biotin blocking kit (Vector Laboratories Inc., Burlingame, UK). The slides were then stained with a rabbit polyclonal anti-SMA antibody (Ab) diluted in PBS containing 0.1% saponin and 10% normal human serum for 1 h at room temperature (2 μg/ml, Abcam, Cambridge, UK). After washes in PBS, slides were incubated with a biotinylated goat anti-rabbit Ab (6.5 μg/ml; Stratech Scientific Unit, Newmarket Suffolk, UK) for 45 min at room temperature. A third layer of soluble complexes of StreptABComplex/HRP (DAKO, Glostrup, Denmark) was incubated for an additional 30 min and developed with peroxidase substrate kit DAB (Vector Laboratories Inc., Burlingame, California, USA). Fibroblasts were counterstained with Harris' hematoxylin (VWR, Leicestershire, UK) and mounted in faramount aqueous mounting medium (DAKO, Glostrup, Denmark). Images were acquired using a Leica TCS SP confocal microscope (Heidelberg, Germany). Substitution of the primary Ab with an irrelevant isotype-matched Ab of the same species was used as a negative control.

### Western blotting

Confluent NHLF were stimulated as before then harvested using RIPA buffer (Invitrogen) following the manufacturer's instructions. Protein concentration was determined using the BCA protein assay (Pierce), against a bovine serum albumin standard curve.

15 μg protein samples were separated on 10% Bis-Tris gels in MOPS SDS Running Buffer (Invitrogen), transferred to polyvinylidene difluoride membrane (Bio-Rad) and probed with a rabbit polyclonal anti-α-SMA Ab (1/1000 dilution; AbCam). Immunoblots were then incubated with peroxidase-conjugated goat anti-rabbit IgG (1/2000 dilution, DakoCytomation) and developed using the ECL + Western blotting detection system (Amersham). Blots were stripped and re-probed with a mouse monoclonal anti-vimentin antibody (1/2000 dilution, Sigma), to ensure equal protein loading.

### Transfection and promoter assays

The connective tissue growth factor (CTGF) promoter- (pCT-sb, 2 μg) Luciferase plasmid and Renilla luciferase control reporter vector (phRL-TK, 5 ng) were transfected into NHLF, seeded in 6-well plates, with PrimeFect I DNA Transfection Reagent (1:10 dilution, Lonza Rockland Inc, Rockland, ME, USA) diluted in serum free FGM. Transfection medium was changed after 24 h to 0.2% FBS containing 5 ng/ml TGF-β1 alone, or 100 ng/ml BMP-4 or BMP-7 alone or 5 ng/ml TGF-β1 and 100 ng/ml BMP-4 or BMP-7. After 24 h, luciferase activity was measured by the dual luciferase assay system (Promega UK, Southampton, UK) according to manufacturer's instruction using a TopCount.NXT microplate luminescence counter (PerkinElmer Life, Milano, Italy). Firefly luciferase activity was normalized by the activity of the Renilla luciferase under the control of thymidine kinase promoter of phRL-TK. Results are given as relative light units. MFB-F11 cells (mouse fibroblasts isolated from *Tgfb1*^*-/- *^mice stably transfected with TGF-β responsive Smad-binding elements coupled to a secreted alkaline phosphatase reporter gene, SBE-SEAP plasmid [34]) were seeded at 4 × 10^4 ^cells/well in 96-well plates. After 4 h in DMEM containing 10% FBS, cells were incubated with TGF-β1 and/or BMP-4 and BMP-7 as described for 24 h in 100 μl of serum free DMEM. All the conditions were tested in duplicate. SEAP activity was measured in 10 μl culture supernatant using Great EscAPe SEAP Reporter System 3 (Clontech Laboratories, Inc., California, USA) according to the manufacturer's instructions with a microplate luminescence counter.

### Statistical analysis

Data were analyzed using Prism 4.0 for Windows (GraphPad Software Inc.) using Friedman test and Wilcoxon post test. The results are expressed as means ± SEM for the indicated number of experiments. The Spearman rank-order method was assessed to determine correlations between the different molecules studied.

## Results

### BMP receptor expression in NHLF

**Figure 1:**
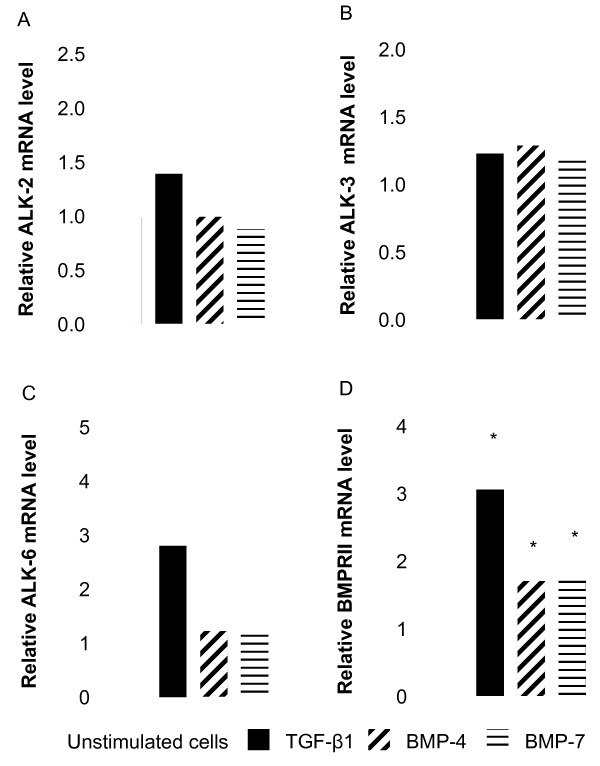
**Effect of TGF-β superfamily members on BMP type I and type II receptor transcript levels**. NHLF were stimulated with 5 ng/ml TGF-β1 or 100 ng/ml BMP-4 or BMP-7 for 24 h. Cells were harvested, RNA extracted and reverse transcribed, and a real-time quantitative PCR for *ALK-2 *(*A*), *ALK-3 *(*B*), *ALK-6 *(*C*), and *BMPRII *(*D*) was performed. Results are expressed as the ratio of each transcript relative to the geometric mean of mRNA expression of the housekeeping genes *UBC*, *SDHA*, and *RPL13a*. Data are mean ± SD of five independent experiments. *, *p *< 0.05, as compared to unstimulated cells.

In order to confirm the ability of NHLF to respond to the BMPs, we determined the basal expression of mRNA encoding the BMP receptors. Unstimulated adult NHLF expressed the BMP type I receptors Activin receptor-like kinase (ALK)-2, ALK-3 and ALK-6 as well as the type II receptor, BMPRII, at the mRNA level as shown in Table 1. The transcripts encoding ALK-2, ALK-3 and ALK-6 were not modulated (Figures 1A, B and 1C) whereas mRNA for BMPRII was significantly up-regulated by TGF-β1, BMP-4 and BMP-7 (Figure. 1D).

### TGF-β superfamily members do not affect NHLF viability and proliferation

**Figure 2:**
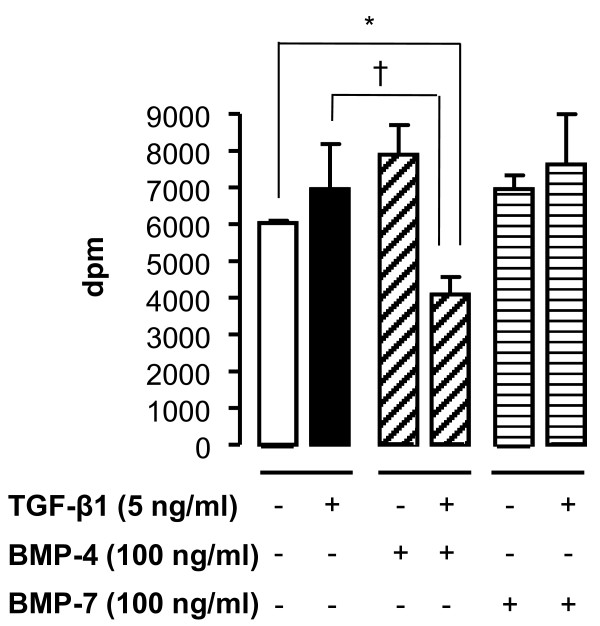
**Simultaneous incubation of NHLF with TGF-β1 and BMP-4 inhibits cell proliferation**. [3H]thymidine incorporation in NHLF in response to tissue culture media with 2% FBS in the presence of 5 ng/ml TGF-β1 or 100 ng/ml BMP-4 or BMP-7 alone or with TGF-β1 in the presence of BMP-4 or BMP-7 for 36 h. [3H]thymidine was added for the last 6 h of incubation. Data are mean ± SD of five independent experiments. *, *p *< 0.05, as compared to unstimulated cells and †, p < 0.05, as compared to TGF-β1-stimulated cells.

Cell viability was determined by MTT assay to verify that the concentrations of TGF-β1 and BMPs used were not toxic to NHLF. None of the conditions tested affected viability of NHLF in FGM media with or without 2% FBS (data not shown). Fibroblast and myofibroblast proliferation and accumulation in the sub-epithelial area is a feature of lung remodelling. Therefore, we determined the effect of TGF-β family members on proliferation of NHLF. TGF-β1, BMP-4 and BMP-7 had no effect on cell proliferation as compared to untreated-cells. However, the addition of BMP-4, but not BMP-7, to TGF-β1-stimulated NHLF led to a significant decrease in cell proliferation as compared to either untreated or TGF-β1-stimulated cells (Figure 2).

### BMP-4, but not BMP-7, downregulates TGF-β1-induced ECM protein expression

**Figure 3:**
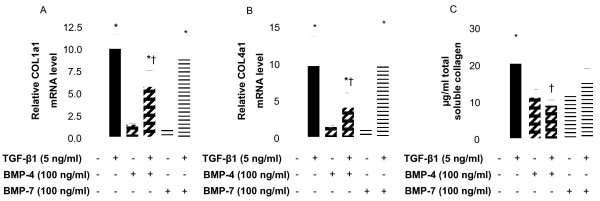
**TGF-β1-induced collagen expression in NHLF is downregulated by BMP-4**. NHLF were stimulated with 5 ng/ml TGF-β1 or 100 ng/ml BMP-4 or BMP-7 alone, or with TGF-β1 in the presence of BMP-4 or BMP-7 for 24 h (A and B) or 72 h (C). Cells were harvested, RNA was extracted, reverse transcribed, and a real-time quantitative PCR for collagen type I alpha 1 chain (*COL1a1, A*) and collagen type IV alpha 1 chain (*COL4a1*, *B*) was performed. Results are expressed as the ratio of each transcript relative to the geometric mean of mRNA expression of the housekeeping genes *UBC*, *SDHA*, and *RPL13a*. Total soluble collagen release was quantified in the cell supernatants by Sircol assay (C). Data are mean ± SD of five independent experiments. *, *p *< 0.05, as compared to unstimulated cells and †, p < 0.05, as compared to TGF-β1-stimulated cells.

**Figure 4:**
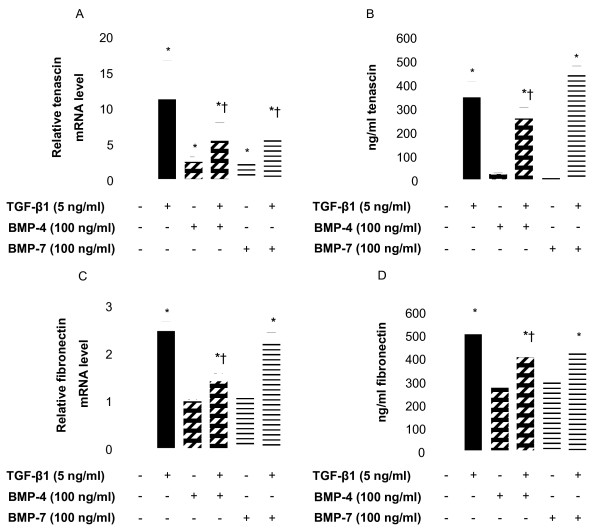
**TGF-β1-induced ECM protein expression in NHLF is down-regulated by BMP-4**. NHLF were stimulated with 5 ng/ml TGF-β1 or 100 ng/ml BMP-4 or BMP-7 alone or with TGF-β1 in the presence of BMP-4 or BMP-7 for 24 h (A and B) or 48 h (C and D). Cells were harvested, RNA was extracted, reverse transcribed, and a real-time quantitative PCR for *tenascin C *(*A*) and *fibronectin *(*C*) was performed. Results are expressed as the ratio of each transcript relative to the geometric mean of mRNA expression of the housekeeping genes *UBC*, *SDHA*, and *RPL13a*. Tenascin C and fibronectin protein were quantified in the cell supernatants by specific ELISAs (B and D, respectively). Data are mean ± SD of five independent experiments. *, *p *< 0.05, as compared to unstimulated cells and †, p < 0.05, as compared to TGF-β1-stimulated cells.

There is extensive published literature describing TGF-β1-driven ECM production in the airways as well as the contribution of fibroblasts to the thickness of the sub-basement membrane, however the role of BMPs in this phenomenon is not yet described in the lung. Incubation of NHLF for 24 h in the presence of 5 ng/ml TGF-β1 significantly up-regulated the expression of mRNAs encoding collagen types I and IV (10- and 9-fold increase, respectively, Figures 3A and 3B). The increase in mRNA transcripts correlated with increased synthesis and release of total soluble collagen measured in cell supernatants (Figure 3C). Transcripts for tenascin C and fibronectin were also upregulated by TGF-β1 (11- and 2.5-fold increase, respectively, Figures 4A and 4C). This increase was reflected at the protein level (18- and 1.7-fold increase, Figures 4B and 4D, respectively), as determined by specific ELISA. In contrast, BMP-4 and BMP-7 (100 ng/ml) did not affect expression of the transcripts encoding collagen type I or IV (Figures 3A and 3B), or fibronectin (Figure 4C). However, a moderate but significant induction of the mRNA for tenascin C was measured after incubation of NHLF with both BMP-4 and BMP-7 (Figure 4A). BMP-4 inhibited the TGF-β1-induced increase in the level of the transcripts encoding collagen type I and IV (Figures 3A and 3B), tenascin and fibronectin (Figures 4A and 4C). A similar effect was observed at the protein level with a 50% decrease in total soluble collagen synthesis (Figure 3C), inhibition of the release of tenascin C and fibronectin (30% and 20%, respectively, Figures 4B and 4D). In contrast, BMP-7 did not modify the TGF-β1-induced up-regulation of the transcripts and proteins examined except for a significant suppression of the expression of mRNA for tenascin C (Figure 4A) but this result was not confirmed at the protein level (Figure 4B).

### TGF-β family members modulate collagenase and gelatinase activities and expression

**Figure 5:**
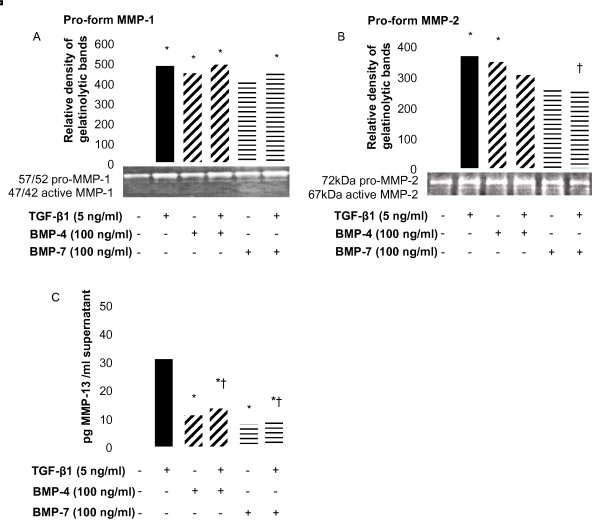
**Effect of TGF-β superfamily members on MMP activity and expression level**. NHLF were stimulated with 5 ng/ml TGF-β1 or 100 ng/ml BMP-4 or BMP-7 alone or with TGF-β1 in the presence of BMP-4 or BMP-7 for 72 h. Cell supernatants were collected to perform zymography (A and B) and ELISA (C). Representative gelatin zymograms and related graphic plot of the bands obtained in zymographs for the pro-forms of MMP-1 (A) and MMP-2 (B) were performed. Gelatinolytic activity of the pro- and active forms of MMP-1 (57/52 and 47/42 kDa) and pro- and active forms of MMP-2 (72 and 67 kDa) are indicated. MMP-13 release was quantified in the cell supernatants by specific ELISA (C). Data are mean ± SD of five independent experiments. *, *p *< 0.05, as compared to unstimulated cells and †, p < 0.05, as compared to TGF-β1-stimulated cells.

The ECM accumulation observed in the asthmatic lung can result from an increase in ECM protein production and/or a deregulation in proMMP activities, the activation of these proenzymes being a critical step that leads to ECM breakdown. NHLF were stimulated for 72 h with either TGF-β1, BMP-4 or BMP-7 or TGF-β1 in combination with BMP-4 or BMP-7, and MMP activity in the cell supernatants was detected on gelatine gels by zymography. Both TGF-β1 and BMP-4 led to a moderate but significant increase in the gelatinolytic activity of the pro-forms of MMP-1 (57 and 52 kDa, Figure 5A) and MMP-2 (72 kDa, Figure 5B) whereas the activity of the active forms was not modulated (47 and 42 kDa for MMP-1 and 67 kDa for MMP-2). BMP-7 itself did not alter the expression of MMP-1 or MMP-2 but its addition to TGF-β1-stimulated cells led to a significant down-regulation in the activity of the pro-MMP-2 as compared to cells stimulated with TGF-β1 alone (Figure 5B). MMP-9 activity was not detected, regardless of the stimulation conditions. MMP-13 release from NHLF was decreased in the presence of BMP-4 and BMP-7 compared to untreated- or TGF-β1-stimulated cells (Figure 5C). The inhibition of MMP-13 release was of similar magnitude when the BMPs were incubated in the presence of TGF-β. Increasing the concentration of BMPs to 1 μg/ml did not result in further MMP-13 reductions (data not shown).

### TGF-β1-induced fibroblast differentiation is partially inhibited by BMP-7

**Figure 6:**
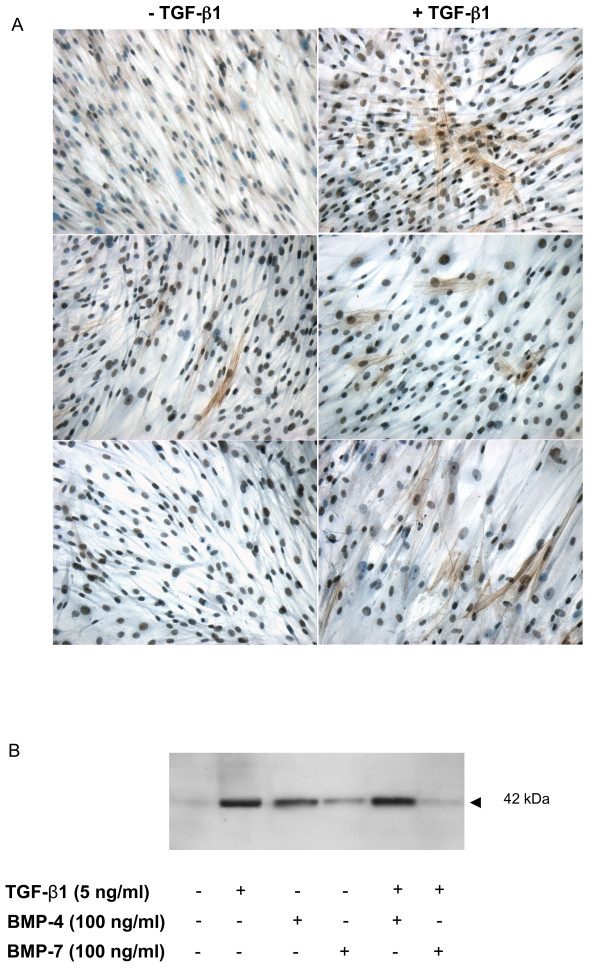
**TGF-β1-induced myofibroblast like phenotype in NHLF is partially inhibited by BMP-7**. NHLF were stimulated with 5 ng/ml TGF-β1 or 100 ng/ml BMP-4 or BMP-7 or with TGF-β1 in the presence of BMP-4 or BMP-7 for 72 h. Representative panel of α-SMA expression was obtained by immunohistochemistry (A) and western blot of cell lysates for α-SMA is shown in (B). Data are representative of five independent experiments.

Fibroblast differentiation into myofibroblasts is crucial in tissue remodelling, wound healing, and various fibrotic disorders in the lung and the contribution of TGF-β to this phenomenon *in vitro *is well documented [5,11,35]. Here we characterized the effect of BMP-4 and BMP-7 on the induction of a myofibroblast-like phenotype in normal lung fibroblasts exposed to TGF-β1. In culture, NHLF basally expressed low levels of αSMA as demonstrated by immunohistochemistry (first panel, Figure 6A). Stimulation with TGF-β1 led to a discernable increase in α-SMA^+ ^cell number (Figure 6B). Western blot of NHLF cell lysates confirmed our observations. Incubation with BMP-4 also led to an increase in the number of αSMA^+ ^cells, whereas BMP-7 alone had no effect (Figure 6A and 6B). BMP-4 did not affect TGF-β1 driven α-SMA expression. In contrast, BMP-7 significantly inhibited TGF-β1 induced differentiation (Figure 6A and 6B).

### BMPs do not affect TGF-β1-induced CTGF promoter and Smad-Binding Element reporter gene activities

**Figure 7:**
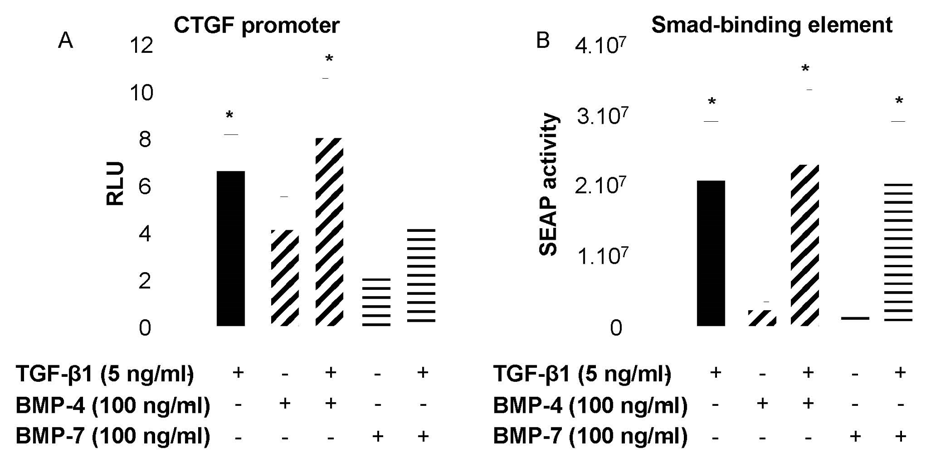
**TGF-β1-induced CTGF promoter and SBE-SEAP reporter activities are not modulated by the BMPs**. (A) The CTGF promoter pCT-sb was transiently transfected into NHLF, cells were then treated with 5 ng/ml TGF-β1 or 100 ng/ml BMP-4 or BMP-7 or with TGF-β1 in the presence of BMP-4 or BMP-7 in FGM containing 0.2% FBS. All assays were performed with 150000 cells/well in 2 ml total volume in 6-well plates and luciferase activity was measured after 24 h induction in 50 μl cell pellet. (B) MFB-F11 cells stably transfected with SBE-SEAP were stimulated with 5 ng/ml TGF-β1 or 100 ng/ml BMP-4 or BMP-7 or with TGF-β1 in the presence of BMP-4 or BMP-7 in serum-free DMEM. All assays were performed with 40000 cells/well in 100 μl total volume in 96-well plates and SEAP activity was measured after 24 h induction in 10 μl supernatant. Data are mean ± SD of five independent experiments. *, *p *< 0.05, as compared with unstimulated cells.

In order to determine the mechanism by which BMPs counteract TGF-β1 effects, activity assays were performed on the CTGF promoter (pCT-sp) transfected in NHLF and TGF-β responsive Smad-binding elements (SBE) reporter gene in the MFB-F11 cell line. TGF-β1 increased luciferase activity in the pCT-sp 6-fold, indicative of CTGF promoter activity (Figure 7A) and SEAP activity in the SBE-SEAP reporter 37-fold (Figure 7B) and this response to TGF-β was not inhibited by either BMP-4 or BMP-7. BMP-4 moderately increased pCT-sp activity (3.6-fold induction, Figure 7A) demonstrating that BMP-4 partially acts via increasing CTGF promoter activity. In contrast, the BMPs had no direct effect on the SBE-SEAP reporter, indicating that they are not able to inhibit binding of phosphorylated Smads (downstream signalling molecules of TGF-β1) to the Smad-Binding Element present on many genes regulated by TGF family members.

## Discussion

In the current study, we determined the ability of two Bone Morphogenetic Proteins, BMP-4 and BMP-7, to modulate the profibrotic effects of TGF-β1 on NHLF. We found that BMP-4 and BMP-7 are able to regulate the synthesis and production of ECM proteins, MMPs and α-SMA in primary lung fibroblasts. BMP-4 inhibits TGF-β1-induced cell proliferation and ECM protein release. Both BMP-4 and BMP-7 decreased MMP-13 release in TGF-β1-stimulated cells. In contrast, only BMP-7 inhibited myofibroblast differentiation and activation of MMP-2 induced by TGF-β1. We have also shown that TGF-β1 can act directly on the BMP pathways by increasing expression of the mRNA encoding ALK-6 and BMPRII.

The ECM is known to be involved in a variety of cellular processes, including morphogenesis, lung remodelling, and modifications in cell shape that occur during differentiation of a number of lung structural cells [5,36]. As a result, changes in the composition of the ECM can profoundly affect the behaviour of cells and lead to airway remodelling in lung fibrotic diseases, including asthma. The increase in ECM deposition results from either increased production or decreased breakdown of matrix products. Deregulation of the proteolytic-antiproteolytic network and inappropriate secretion of various MMPs by stimulated lung structural cells is thought to be involved in the pathophysiology of asthma [37]. The contribution of TGF-β1 to ECM accumulation, and to fibroblast differentiation and proliferation has been widely reported [5,35,38,39]. Its action is mainly driven by activation of CTGF, resulting in stimulation of fibroblast proliferation, myofibroblast differentiation and collagen synthesis [40,41]. In this study, we confirmed the ability of TGF-β1 to induce production of the ECM proteins collagen types I and IV, fibronectin and tenascin C, and to induce myofibroblastic differentiation. However, we did not observe TGF-β1-induced fibroblast proliferation as previously reported by some groups [9,42,43] but those data might be considered controversial since the effect of TGF-β1 on fibroblast proliferation is dependent on its concentration [44]. The increased expression of αSMA correlates with the release of collagen and activation of MMP-1, the major enzyme involved in degradation of native collagen, which is in accordance with the data showing that myofibroblasts are the major source of collagen type I in the lung [45]. Finally we confirmed the ability of TGF-β1 to activate both the CTGF promoter and Smad-binding elements (SBE) contained in the promoter region of more than 500 target genes responding to TGF-β1 [34].

In most models and cell types, BMP-7 opposes TGF-β1-mediated ECM protein production *in vivo *and *in vitro *[19-26]. BMP-7 regulates the ECM breakdown in human chondrocytes by downregulating MMP-13 [46]. Nevertheless, two recent studies have shown that BMP-7 fails to inhibit TGF-β mediated fibrosis in the lung, skin and renal tubular epithelial cells [27,28]. In our model, BMP-7 did not counteract the increase in ECM proteins induced by TGF-β1. However, we have shown for the first time in lung fibroblasts that BMP-7 reduces not only the basal fibroblast-related expression of MMP-13 but also the induced expression of this protein following stimulation by TGF-β1. MMP-13, an interstitial collagenase, is the principal enzyme involved in the initiation of collagen breakdown. MMP-2 can serve as an activator of other MMPs, namely MMP-13 [47]. Thus, the downregulation of TGF-β1-induced MMP-2 activity by BMP-7 is in accordance with the inhibition shown for MMP-13. BMP-7 could contribute to a reduction in airway remodelling by inhibiting some MMPs without affecting ECM protein release. BMP-7 was also able to counteract TGF-β1-induced fibroblast differentiation. This potential regulatory function of BMP-7 confirms its ability to contribute to resolution of lung remodelling since increased numbers of myofibroblasts and fibroblast differentiation are major features of airway remodelling.

The role of BMP-4 in degradation and remodelling of the ECM remains unclear, particularly in the lung. In fact, little is known about the properties of BMP-4 either *in vivo *or *in vitro *in the lung or other tissues. A regulatory effect of BMP-4 on MMP-13 release in human adipocytes has been reported [48] as well as an inhibition of cell proliferation and an upregulation of αSMA expression in foetal lung fibroblasts [30], but nothing is known of its effects on adult lung fibroblasts. Here, we demonstrate for the first time that BMP-4 is able to counteract the increase in ECM protein release induced by TGF-β1 in NHLF. We also reported that BMP-4 not only reduces the basal fibroblast-related expression of MMP-13 but also its expression induced by TGF-β1. The contribution of BMP-4 to the reduction of airway remodelling could result from a direct modulation of the production of ECM proteins as well as MMP-13. In our study, BMP-4 had no direct effect on fibroblast proliferation. This is in contrast to the study of Jeffery *et al. *which reported inhibition of fibroblast proliferation but their study was performed on foetal fibroblasts which possess a higher intrinsic capacity for self-renewal than adult cells. The differential response of NHLF to BMP-4 and BMP-7 may also be a function of the signalling pathways utilized or, alternatively, the regulation of different transcriptional repressors or activators. It is likely that BMP-4 and BMP-7 act via different pathways to regulate ECM accumulation. BMP-7 selectively binds to receptors distinct from those of BMP-4: BMP-4 binds and activates ALK-3 and ALK-6 whereas BMP-7 preferentially binds to ALK-2 and ALK-6 [49-51]. Furthermore, the actions of the BMPs, at least BMP-7, may be tissue or cell type specific since the inhibitory effects of BMP-7 on remodelling are less pronounced in the lung than other tissues.

## Conclusions

Evidence from animal models suggests that airway remodelling in asthma may be prevented or reversed using agents which target TGF-β [8,52]. Therefore, modulation of TGF-β or its activity represents a potential therapeutic target for asthma and other fibrotic diseases. We were the first to report dysregulation of BMP and BMPR expression in asthma [31]. Others have shown an up-regulation of Gremlin, an inhibitor of BMP-4 signaling pathways, in idiopathic pulmonary fibrosis and have suggested that this increased expression of Gremlin may be a key event in the persistence of myofibroblasts in the lung interstitium [53]. Taken together, these data lend weight to the argument that BMP-4 plays a crucial role in the regulation of lung fibroblasts in disease. Our current study has determined that BMP-7 can also exert some functional effects on TGF-β1-driven profibrotic processes in normal lung fibroblasts. These BMPs appear to be attractive targets for therapeutic intervention in asthmatic disease although the blockade of TGF-β1 by only one of these molecules may not be sufficient to totally inhibit activity. A better understanding of how BMPs act *in vitro *on lung structural cells and *in vivo *in animal models of asthma could potentially lead to the amelioration of airway remodelling and consequently a decrease of asthma symptoms.

## Competing interests

The authors declare that they have no competing interests.

## Authors' contributions

SP carried out the majority of experimental work and drafted the manuscript. GAC carried out the western blotting. ABK participated in the design and coordination of the study. CML conceived of the study, participated in its design and coordination and helped to draft the manuscript. All authors read and approved the final manuscript.
